# Gut Microbiome Alters the Activity of Liver Cytochromes P450 in Mice With Sex-Dependent Differences

**DOI:** 10.3389/fphar.2020.01303

**Published:** 2020-10-02

**Authors:** Lenka Jourová, Marketa Vavreckova, Nina Zemanova, Pavel Anzenbacher, Katerina Langova, Petra Hermanova, Tomas Hudcovic, Eva Anzenbacherova

**Affiliations:** ^1^Department of Medical Chemistry and Biochemistry, Faculty of Medicine and Dentistry, Palacky University, Olomouc, Czechia; ^2^Department of Pharmacology, Faculty of Medicine and Dentistry, Palacky University, Olomouc, Czechia; ^3^Department of Medical Biophysics, Faculty of Medicine and Dentistry, Palacky University, Olomouc, Czechia; ^4^Institute of Microbiology, Czech Academy of Sciences, Novy Hradek, Czechia

**Keywords:** liver cytochromes P450, gut microbiome, sex difference, germ-free mice, metabolism of drugs

## Abstract

Sexual differences and the composition/function of the gut microbiome are not considered the most important players in the drug metabolism field; however, from the recent data it is obvious that they may significantly affect the response of the patient to therapy. Here, we evaluated the effect of microbial colonization and sex differences on mRNA expression and the enzymatic activity of hepatic cytochromes P450 (CYPs) in germ-free (GF) mice, lacking the intestinal flora, and control specific-pathogen-free (SPF) mice. We observed a significant increase in the expression of *Cyp3a11* in female SPF mice compared to the male group. However, the sex differences were erased in GF mice, and the expression of *Cyp3a11* was about the same in both sexes. We have also found higher *Cyp2c38* gene expression in female mice compared to male mice in both the SPF and GF groups. Moreover, these changes were confirmed at the level of enzymatic activity, where the female mice exhibit higher levels of functional CYP2C than males in both groups. Interestingly, we observed the same trend as with CYP3A enzymes: a diminished difference between the sexes in GF mice. The presented data indicate that the mouse gut microbiome plays an important role in sustaining sexual dimorphism in terms of hepatic gene expression and metabolism.

## Introduction

The gut microbiome, an aggregate genome of trillions of microorganisms, provides a wide range of beneficial functions for the host and has an immense effect on the host’s health status and predisposition to disease (Kinross et al., 2011). This ecosystem of bacteria, archaea, viruses, and unicellular eukaryotes is mostly stable in the long run, but transiently they may be affected by many factors (Schlomann and Parthasarathy, 2019). Among the wide range of factors that influence the composition of the gut microbiota, diet seems to be the most potent (Wilson et al., 2020). Although the effect of sex appears to be less influential, some studies highlight sex-based differences in the gut microbiome (Markle et al., 2013; Haro et al., 2016; Org et al., 2016). Also, the interactions between the diet and gut microbiota tended to be sex-dependent (Bolnick et al., 2014). It is now clear that without this bacterial community, the immune system and other physiological processes would never reach their full potential (Tlaskalová-Hogenová et al., 1983), and an imbalance in its composition leads to a broad spectrum of pathological conditions. Moreover, studies have shown that the gut microbiota influence the metabolism and toxicity of xenobiotics (Sousa et al., 2008; Jourova et al., 2016; Wilson and Nicholson, 2017) with an impact on their oral bioavailability and pharmacokinetics (Yoo et al., 2014; Jourova et al., 2019).

Cytochromes P450 (CYPs) are key enzymes involved in the initial metabolism of drugs in humans, including 70–80% of all drugs in clinical use (Anzenbacher and Anzenbacherová, 2001). The expression of these enzymes is under the control of specific nuclear receptors (Hakkola et al., 2018), and their regulation is influenced by many factors such as genetic polymorphisms, regulation by cytokines, pathological states, age, etc. (Zanger and Schwab, 2013). In addition, the gut microbiome mentioned above has been shown to play an important role in this, and its effect on the expression of CYP enzymes has been published recently (Matuskova et al., 2010; Selwyn et al., 2015; Selwyn et al., 2016; Jourova et al., 2017). Further, sex influences a number of important pharmacokinetic parameters including the expression of drug-metabolizing enzymes and transporters (Yang et al., 2012). It has been also found that sex differences together with circadian rhythmicity influence the CYPs and nuclear receptor expression in the mouse liver (Lu et al., 2013).

Sexual differences and the composition/function of the gut microbiome are not considered the most important players in the drug metabolism field; however, from the recent data it is obvious that they may significantly affect the patient’s response to therapy. Deeper knowledge of their combined effect on the function of CYPs may be helpful for predicting pharmacokinetics and drug response in a particular patient and avoiding undesirable side effects, which is especially important for drugs with a narrow therapeutic index.

Here, we evaluated the effect of microbial colonization and sex differences on the mRNA expression and enzymatic activity of hepatic cytochromes P450 (CYPs) in germ-free (GF) mice, lacking the intestinal flora, and control specific-pathogen-free (SPF) mice.

## Materials and Methods

### Chemicals

Protease inhibitor cocktail tablets (EDTA free Complete Protease Inhibitor Cocktail Tablets) were supplied by Roche (Prague, Roche, Czech Republic). Hydrochloric acid (p.a., 37%) was supplied by Penta (Prague, Czech Republic), and sodium chloride and dimethyl sulfoxide were obtained from Lach-Ner (Neratovice, Czech Republic). Acetonitrile was supplied by VWR International (Prague, Czech Republic) and was obtained in the highest purity available.

Substrates of orthologous human CYP forms for the determination of murine CYP activities (ethoxyresorufin, diclofenac, diazepam, bufuralol) and their respective metabolites were supplied by Sigma-Aldrich CZ (Prague, Czech Republic). Midazolam was purchased from Abcam (Cambridge, UK). Other chemicals were purchased from Sigma-Aldrich CZ (Prague, Czech Republic). All chemicals were of the highest purity available.

### Animals and Experimental Design

Male and female germ-free (GF) and specific-pathogen-free (SPF) inbred BALB/c two-month-old mice were used for the experiment (5 animals per group). GF mice were born and housed under sterile conditions in Trexler-type plastic isolators and fed a 50 kGy irradiated sterile pellet diet of Altromin 1410 (Altromin, Lage, Germany) and sterile water *ad libitum*. Axenicity was assessed every two weeks by confirming the absence of bacteria, moulds, and yeast by aerobic and anaerobic cultivation of mouse feces and swabs from the isolators in VL (Viande-Levure), Sabouraud-dextrose, and meat-peptone broth and subsequent plating on blood, Sabouraud, and VL agar plates. SPF mice were kept in IVC cages (Tecniplast, Italy) and fed with the same sterile diet as their gnotobiotic counterparts. SPF mice were regularly checked for the absence of potential pathogens according to an internationally established standard (FELASA). Animals were kept in a room with a 12 h light-dark cycle at 22^o^C. Two-month-old mice were used for our experiments.

The mice were euthanized at the age of 8–12 weeks by cervical dislocation and exsanguination. The livers were aseptically removed, weighed, frozen in liquid nitrogen, and subsequently stored at -70°C until further processing.

### Preparation of Subcellular Fractions

Microsomal fractions were obtained from the liquid nitrogen-frozen liver of mice. Microsomes were prepared by differential centrifugation according to established protocols (Lake, 1987). The buffer for homogenization was supplemented with protease inhibitor cocktail tablets (Roche CZ, Prague). Microsomal fractions were stored at −80°C. Protein concentrations in the microsomal fractions were assayed using a bicinchonic acid assay according to the established method (Smith et al., 1985). The concentration of CYP enzymes in liver microsomes was determined using difference spectroscopy (Zhang et al., 2010).

### RNA Isolation and Quantitative Real-Time PCR (qPCR)

Total RNA was isolated from tissue samples stored in RNAlater (Qiagen, Dynex, Czech Republic) using an RNeasy Plus Mini Kit (Qiagen). RNA concentration and purity was determined spectrophotometrically, and the RNA integrity was verified by gel electrophoresis. First strand cDNA was synthesized from total RNA with a Transcriptor High-Fidelity cDNA synthesis kit (Roche, Prague, Czech Republic). Real-time PCR for CYPs quantification was performed in a LightCycler 1536 Instrument (Roche, Prague, Czech Republic) using specific TaqMan Gene Expression Assays (Applied Biosystems, Life Technologies, Prague, Czech Republic). The 1536-well plates were pipetted using an Automate Labcyte Echo (Dublin, Ireland).

The calculations were based on the “Delta-Delta Ct method” (Livak and Schmittgen, 2001). The data was expressed as the fold change in the treatment groups relative to the control, and HPRT1 and 18S RNA were used as an internal control.

### Enzyme Assays

Enzyme activities were assayed in the microsomal fractions from the homogenate of mouse liver. The amount of organic solvents in the final reaction mixtures did not exceed 1% (v/v). The enzyme activities of selected CYP enzymes were measured according to the established method (Kronbach et al., 1989; Phillips and Shephard, 2006). For the determination of murine CYP activities, substrates of orthologous human CYP forms were used: CYP1A1/2, ethoxyresorufin (murine CYP1A2); CYP2C9, diclofenac (murine 2C subfamily); CYP2C19, diazepam (murine 2C subfamily); CYP2D6, bufuralol (murine 2D22) and CYP3A4, midazolam (murine CYP3A11 and 3A13). All activities were measured using a Shimadzu LC-20 HPLC system (Shimadzu, Kyoto, Japan) with UV or fluorescence detection being used for the determination of metabolites. The measurements were performed in a LiChrospher RP-18 column or a Chromolith® High Resolution RP-18 endcapped column (determination of midazolam substrate) (Merck, Germany).

### Statistics

The normal distribution of data was tested using Shapiro-Wilk test. The statistical significance of gene expression was determined by two-way ANOVA using software IBM SPSS Statistics for Windows, Version 23.0 (Armonk, NY: IBM Corp.). Differences were regarded as statistically significant when the p-value was lower than 0.05. Software GraphPad Prism 8 (GraphPad Software Inc., California, USA) was used to create the graphs. Due to the scarcity of material, the statistical significance of activity assays could not be determined.

## Results

We investigated the role of sex and presence/absence of the gut microbiome on the gene expression and enzymatic activity of CYPs in the liver using germ-free (GF) mice, lacking the intestinal flora and specific-pathogen-free (SPF) mice. SPF male data were used as a control group.

**Figure 1:**
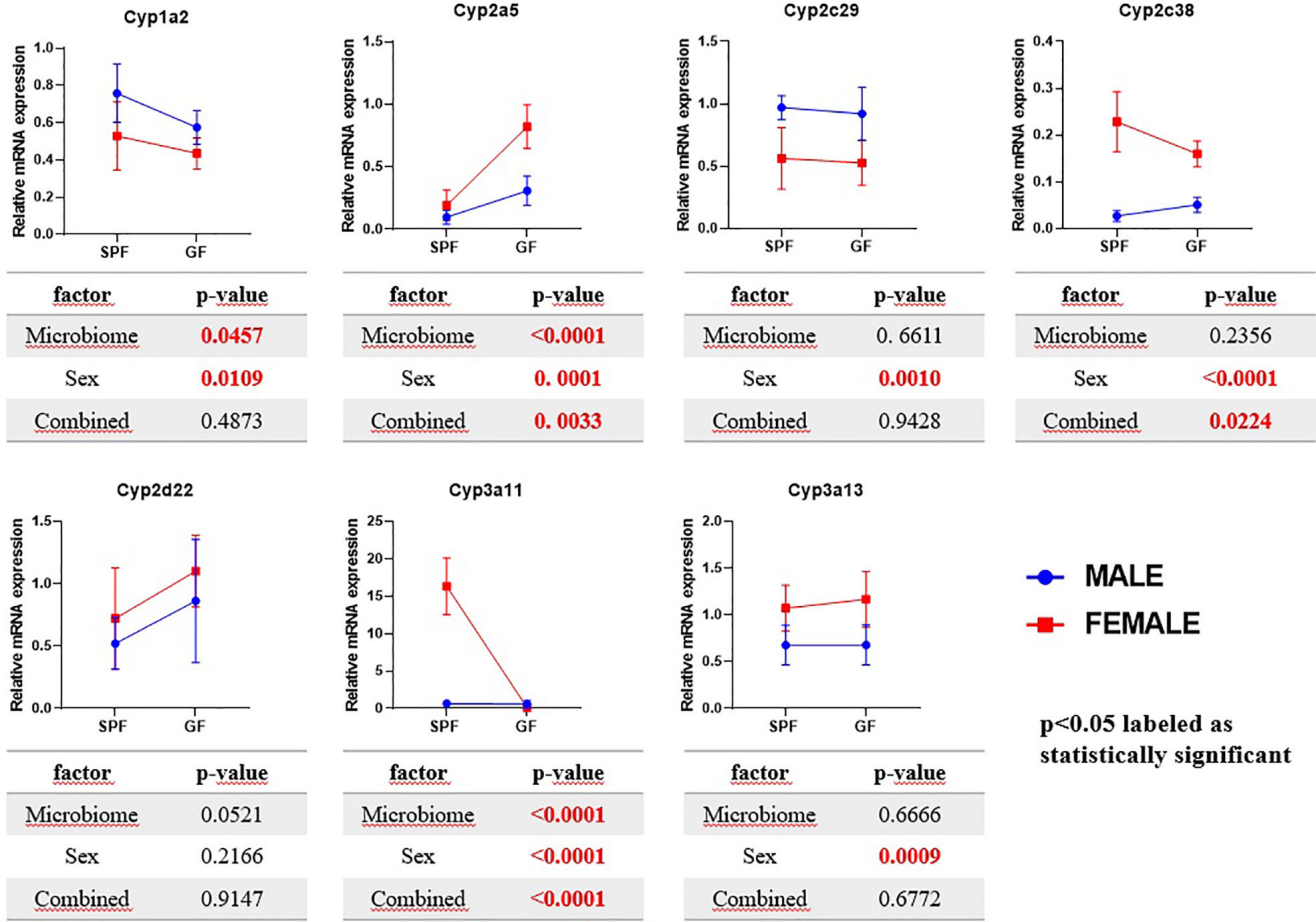
Comparison of estimated marginal mean values of mRNA expression of selected Cyps in relation with microbiome presence and sex in mice liver. The data represent the mean ±SD from 4–5 individual animals. The statistical significance was determined using two-way ANOVA and p-values for the effect of microbiome (i.e., difference between SPF and GF groups), sex and combined effect of the both are shown in the tables below the respective graphs.

The *Cyp3a11* mRNA was significantly increased (21 times) in female SPF mice compared to their male counterparts. Interestingly, these phenomena were not observed in GF mice, where the GF female mice have shown rather decreasing tendency compared to other groups (**Figure 1**).

The expression of *Cyp3a13* was slightly increased in the females of both the group SPF and GF mice. The Cyp2a5 mRNA expression was significantly increased in GF female mice, but not the SPF female group, compared to control. Moreover, the expression of the *Cyp2c38* gene was significantly increased in the females in both groups, GF and SPF, but to a larger extent in SPF mice. In the case of the *Cyp1a2* and *Cyp2c29*, the mRNA expression was significantly decreased in SPF and GF female mice compared to the control (**Figure 1**).

The females of both groups (GF and SPF) exhibited a much greater enzymatic activity of 2C (using diclofenac as substrate) than the male groups (**Table 1**). The effect of gut microbiome was also observed as the GF male and female have shown increased 2C enzymatic activity compared to their SPF counterparts (**Table 1**). The enzymatic activity of CYP3A was significantly decreased in GF mice compared to SPF mice. In the female groups, the activity of CYP3A did not differ significantly from the male groups but had a tendency to decrease (**Table 1**). The other selected CYP activities were not significantly changed by the gut microbiome presence or sex (**Table 1**).

**Table 1:** Difference in enzymatic activity of CYPs in liver of SPF and GF male and female mice.

	SPF		GF	
	Male (CONTROL)	Female	Male	Female
**CYP1A1/2-like**	1.000 ± 0.023	0.868 ± 0.057	1.229 ± 0.010	1.124 ± 0.045
**CYP2C9-like**	1.000 ± 0.017	**6.254 ± 0.086 ▲**	**3.076 ± 0.081 ▲**	**9.950 ± 0.010 ▲**
**CYP2C19-like**	1.000 ± 0.016	1.067 ± 0.011	0.740 ± 0.035	0.624 ± 0.006
**CYP2D6-like**	1.000 ± 0.025	1.126 ± 0.035	0.947± 0.011	1.186 ± 0.009
**CYP3A4-like**	1.000 ± 0.066	**0.710 ± 0.040 ▼**	**0.300 ± 0.092▼**	**0.242 ± 0.046 ▼**

Samples were measured in triplicates in a pooled liver microsomal fraction from 5 animals. Data represent the mean ± SD and values significantly increased (▲) or decreased (▼) relative to the control (SPF male mice) are labelled. Significantly changed values are highlighted in bold.

## Discussion

Recent studies clearly show that the gut microbiota plays an indispensable role in the equation leading to interindividual variation in response to therapy. For this reason, to reach the desired goal of “personalized medicine,” in addition to other factors, we have to take into account our 100 trillion bacterial inhabitants and their genomes with substantial metabolic potential.

The effect of the gut microbiota on the hepatic gene expression of some CYPs in mice has been previously studied (Claus et al., 2011; Selwyn et al., 2015; Selwyn et al., 2016; Jourova et al., 2017). Most of these experiments, however, were only done with male mice and did not consider sex-specific differences. However, sex differences were among the main variables influencing the hepatic drug metabolism in rodents (Corton et al., 2012). Moreover, some animal and human studies pointed out sex/gender-related differences in gut microbiota composition (Markle et al., 2013; Haro et al., 2016; Org et al., 2016). Little is known about the combined effect of both factors. Thus, we assumed not only the effect of the microbiome but also sex differences in the presence/absence of gut microbiota on the regulation of liver biotransformation enzymes.

In rodents, sex differences are one of the most important factors influencing drug metabolism in the liver (Corton et al., 2012). Further, mice females have been shown to have a higher expression of most Cyp genes (Renaud et al., 2011; Lu et al., 2013). A higher expression of *Cyp3a11* in female mice than males was reported earlier (Hernandez et al., 2009; Renaud et al., 2011). Even though in humans the differences are subtler, it is known that the CYP3A4 activity features apparent sexual dimorphism with higher activity in women due to differences in growth hormone patterns (Waxman and O’Connor, 2006). In line with these data, we observed a significant increase in the expression of *Cyp3a11* in female SPF mice compared to the male group. Interestingly, the sex differences were erased in GF mice, and the expression of *Cyp3a11* was about the same in both sexes. In other words, there is significant interaction between the gender and presence of the gut microbiome, which together influence the Cyp3a11 mRNA expression. A recent paper has proposed a possible explanation, showing that the mouse microbiome is required for sex-specific diurnal rhythms of gene expression and metabolism (Weger et al., 2019). The enzymatic activity of CYP3A (using midazolam as a substrate) in both the male and female mice lacking the microbiota was significantly decreased compared to their SPF counterparts. These results are in line with our previous study on the GF and SPF male mice (Jourova et al., 2017). Further we have found that the activity of CYP3A did not differ significantly in female groups from the male groups, but had a tendency to decrease. This data suggests that the down-regulation of CYP3A enzymes may not be sex-specific and, above all, that the gut microbiome is crucial in the synthesis of CYP3A in both sexes.

Moreover, we have found a higher *Cyp2c38* mRNA expression in female mice compared to male mice in both SPF and GF groups. Moreover, these changes were confirmed at the level of enzymatic activity (using diclofenac as substrate), when female mice exhibit higher levels of functional CYP2C than males in both mice groups. Interestingly, we observed the same trend as with CYP3A enzymes: diminishing the changes between sexes in GF mice. The increase in *Cyp2c38* expression in the female group compared to the male was almost two times lower in GF mice than in SPF mice (2 times in the case of enzymatic activity of CYP2C). In other words, sex differences in *Cyp2c38* expression and the enzymatic activity of CYP2C were less pronounced in GF mice. Therefore, not only the effect of sex but also the combined effect of gut microbiome and sex was found significant in regulation of *Cyp2c38* mRNA expression in the liver of mice. In humans, CYP2C and CYP3A enzymes are abundantly present in the liver and, completely or partially, metabolize a large fraction of all prescribed drugs (Anzenbacher and Anzenbacherová, 2001). The presented data thus highlight the possible modulation of the response to a broad spectrum of clinically used drugs by the interaction of gut microbiota and the sex-based hormonal level.

As mentioned in the Introduction, the regulation of the CYP expression is very complex and many factors play a role here (Zanger and Schwab, 2013), and among them, the gut microbiome is gaining the increasing attention of scientists. After all, the gastrointestinal tract, with 100 trillion microbes, is a rich resource of microbial metabolites that may be responsible for the indirect effect of gut microbiome on whole-body metabolism (Lee and Hase, 2014). Moreover, novel potential mechanism of action of the gut microbiome in regulation of host metabolism was defined recently by Weger et al. (2019). They have found that the absence of the microbiome reduces liver sexual dimorphism. In other words, microbial metabolites contribute significantly to maintaining the sex differences in gene expression and metabolism by promoting proper sexual development and growth hormone secretion (Weger et al., 2019). These effects are often associated with activation of nuclear receptors such as aryl hydrocarbon receptor (AhR) and pregnane X receptor (PXR), which participate in regulation of CYPs expression (Zhang et al., 2010; Huang et al., 2016). CYP enzymes, however, are not only involved in detoxification of xenobiotics, but they are involved as well in the synthesis of steroid hormones, eicosanoids, prostaglandins, and thromboxanes (Nebert and Russell, 2002). CYP3A4 contributes to the oxidation of many steroids (Niwa et al., 2015), and its activity in humans is sexually dimorphic (Lamba et al., 2010). The exact mechanism for sex differences in CYP3A4 (and other CYPs) remains elusive but may include altered growth hormone signalling (Waxman and Holloway, 2009). All data mentioned above open the possibility of an important involvement of microbiome-derived metabolites in the signalling pathways regulating hepatic drug metabolism and sexual dimorphism in gene expression.

In conclusion, the current data and the literature indicate that the gut microbiome plays an important role in sustaining the sexual dimorphism in hepatic gene expression and metabolism. As the concept of modulating the gut microbiome to improve health may provide promising therapeutic effects, further studies are needed to explain in more detail the role of the gut microbiota, along with other factors, in the pathways that participate in the regulation of CYP synthesis and drug metabolism. Better understanding the ways gut microbiota may influence drug metabolism could make a significant contribution to the improvement of pharmacotherapy (e.g., in better efficacy and in avoiding the undesirable side effects).

## Data Availability Statement

The raw data supporting the conclusions of this article will be made available by the authors, without reservation, to any qualified researcher.

## Ethics Statement

The animal study was reviewed and approved by The experiment was carried out in accordance with Czech Act No. 359/2012 Coll. for the protection of animals against abuse. All procedures with animals were approved by the Ethics Committee, Ministry of Education of the Czech Republic. Experiments were approved by the Committee for the Protection and Use of Experimental Animals of the Institute of Microbiology. v.v.i., Academy of Sciences of the Czech Republic (approval ID: 50/2011).

## Author Contributions

LJ measured and analyzed the data and, with the help and supervision of EA and PA, wrote the paper. MV measured the enzymatic activity. NZ measured the gene expression. KL performed the statistical analysis. PH and TH performed the experiment with germ-free mice. All authors contributed to the article and approved the submitted version.

## Funding

This work was supported by The Czech Science Foundation (grant no. 19-08294S) and by project Toxicology CZ.02.2.69/0.0/0.0/16_018/0002311.

## Conflict of Interest

The authors declare that the research was conducted in the absence of any commercial or financial relationships that could be construed as a potential conflict of interest.
